# Turning Away From Averted Gazes: The Effect of Social Exclusion on Gaze Cueing

**DOI:** 10.3389/fpsyg.2019.01000

**Published:** 2019-05-16

**Authors:** Roberta Capellini, Paolo Riva, Paola Ricciardelli, Simona Sacchi

**Affiliations:** ^1^ Department of Psychology, University of Milano-Bicocca, Milan, Italy; ^2^ NeuroMI, Milan Center for Neuroscience, Milan, Italy

**Keywords:** social exclusion, averted gaze, social attention, reaffiliation, cueing effect

## Abstract

Past studies showed increased sensitivity to other people’s gaze after social exclusion. In the present research, across two studies, we tested whether social exclusion could affect the basic cognitive phenomenon of gaze-cueing effect, namely, the tendency to redirect visual attention to the same location that other people are looking at. To this purpose, participants were socially excluded or included using the *Cyberball* manipulation. In Study 1, after the manipulation, participants performed a gaze-cueing task in which an individual’s gaze, oriented rightward or leftward, preceded a peripheral target stimulus requiring a simple categorization response. The gaze direction could be congruent or incongruent with the location of the target. Results revealed a reduced gaze-cueing effect for socially excluded than for socially included participants. In Study 2, where human gazes were replaced by arrow cues, such an interaction between social exclusion and trial congruency disappeared, indicating a specific effect of social stimuli. We interpreted these findings with the notion that excluded participants can perceive an averted gaze as a further sign of social exclusion, thus showing a reduced gaze-cueing effect.

Social exclusion has been defined as the experience of being kept apart from others physically (e.g., social isolation) or emotionally (e.g., being ignored or told one is not wanted; Riva and Eck, 2016). As a common phenomenon, social exclusion can take many forms, such as ostracism (Williams, 2007), rejection (e.g., Maner et al., 2007), and discrimination (e.g., Richman et al., 2016). In our everyday life, the experience of being socially excluded can occur in many occasions; from ostracism on the playground to being bullied in the classroom, discriminated at the workplace, and isolated in the later stage of life.

Considering that social exclusion threatens a fundamental psychological need (i.e., the need to belong; Baumeister and Leary, 1995), it has a profound impact on the human mind, affecting the emotional, behavioral, and cognitive levels (Williams, 2009; Riva and Eck, 2016; for a meta-analysis, see Gerber and Wheeler, 2009). From an emotional perspective, instances of social exclusion have been linked with negative affect, including a cluster of emotions such as sadness, anger, anxiety, and feelings of depression (Buckley et al., 2004; Riva et al., 2017). In behavioral terms, previous research suggested that excluded individuals are more prone to self-defeating behaviors including aggression (Twenge et al., 2001), risk-taking (Twenge et al., 2002; Peake et al., 2013; Svetieva et al., 2016; Buelow and Wirth, 2017), and gambling (Pancani et al., 2018). At a cognitive level, social exclusion negatively influences performance on intelligence tests (Baumeister et al., 2002). Moreover, it has been shown that social exclusion can undermine self-regulation abilities (Baumeister et al., 2005; Stenseng et al., 2015) and impair inhibitory control (Leary et al., 2006). Since the self-regulation of exclusion-related distress deploys attentional resources (Chester and DeWall, 2014; Riva et al., 2014a) leaving limited resources for effective inhibitory control (Lurquin et al., 2014), it could lead to more impulsive or prepotent behaviors. For instance, social exclusion can increase aggression and decrease prosocial behavior (Twenge et al., 2001, 2007) and foster consumption of chocolate chip cookies (Baumeister et al., 2005). Importantly, social exclusion seems to affect not only late-stage cognitive processes but also early-stage cognitive processes, such as selective attention (Xu et al., 2017), which represents an important component of inhibitory control (Friedman and Miyake, 2004; Diamond, 2013). Recently, Xu et al. (2017) showed that exclusion influences selective attention by impairing distractor suppression; in a visual search task, they presented target (as inclusion-related cues) and distractor (as exclusion-related cues) stimuli and found behavioral and neural evidence that exclusion exerted different impacts on target and distractor processing. Only excluded participants reported smaller distractor-positivity amplitudes, as a reflection of distractor suppression, whereas both excluded and included reported similar target-negativity amplitudes as a reflection of target enhancement. Thus, the influence of social exclusion on selective attention was driven by distractor suppression but not by target enhancement.

In parallel, the experience of being excluded may also enhance sensitivity to affiliative social cues (Pickett et al., 2004; see also Shilling and Brown, 2016). Prior study illustrated that ostracized participants, compared to included and control participants, were more accurate at discriminating between genuine and fake smiles (Bernstein et al., 2008) and better able to differentiate between happy and angry faces relative to their ability to differentiate within happy and angry face categories (Sacco et al., 2011). DeWall et al. (2009) showed that socially excluded individuals displayed an attentional bias toward affiliative cues. For instance, they found that participants expecting exclusion were more sensitive to searching for emotional faces in a crowd of neutral faces, more fixated on smiling faces, and slower at disengaging from smiling faces. A similar pattern has been revealed in participants excluded from a virtual ball-tossing game (Xu et al., 2015). Also, Chen et al. (2017b) demonstrated that social exclusion increases attention toward social cues, especially positive social cues.

Accordingly, a great deal of research suggested that excluded individuals have stronger motivation in forming new relationships and are more willing to affiliate with those who may signal approachable intentions toward them (Maner et al., 2007); thus, they may imitate others’ movements (Lakin et al., 2008), conform (Williams et al., 2000), and obey (Riva et al., 2014b) to a greater extent than non-excluded individuals. Furthermore, successful reaffiliation can reduce the unpleasant effects of ostracism (DeWall et al., 2010). On the other hand, when reconnection is not possible, people might be—as recovery strategies from social exclusion—motivated to stop their negative emotional state (Buckley et al., 2004) through emotion regulation processes (Riva, 2016), for instance, by turning attention away from their exclusionary situation (i.e., distraction; Wesselmann et al., 2013). Therefore, when there are no reaffiliation opportunities, excluded individuals may sometimes withdraw from interactions and search for self-isolation (Romero-Canyas et al., 2010; Cuadrado et al., 2015), and start to perceive other people as particularly unfriendly and unapproachable (Richman et al., 2016).

## The Role of Direct and Averted Gaze in Socially Excluded Individuals

Extensive research over the past decade suggests that the eyes convey a wealth of personal information and about their direction of attention to specific people, places, and objects (Birmingham and Kingstone, 2009). Eye gaze, especially direct gaze (i.e., when the gaze of another individual is directed at the observer), is used to indicate interest, express closeness, regulate interactions (Conty et al., 2016), and conveys social connection (Wirth et al., 2010; Wesselmann et al., 2012).

Moreover, eye contact has powerful effects on the receiver (for reviews, see Kleinke, 1986; Hietanen, 2018). Seeing another person with direct gaze automatically elicits positive affective reactions in the observer (Chen et al., 2016, 2017a), increases autonomic arousal (Helminen et al., 2011), and activates brain responses indicative of a tendency to approach (Hietanen et al., 2008). Interestingly, such effects of eye gaze do not have to be conscious (Burra et al., 2013). On the other hand, an averted eye gaze has been deemed to represent a common sign of social exclusion (Williams et al., 1998). Being denied others’ direct gaze can elicit brain mechanisms related to avoiding motivation (Hietanen et al., 2008; Abeles and Yuval-Greenberg, 2017) and increase the willingness to act aggressively toward the interaction partner (Wirth et al., 2010). Supporting this notion, a study found that receiving averted gaze, compared to receiving direct gaze, leads participants to feel ostracized (Wirth et al., 2010). In this study, participants briefly watched a face on a computer screen portraying either direct (i.e., by looking at the participant) or averted (i.e., by looking left and right, but not at the participant) gaze. Results showed that even briefly exposing participants to averted gaze, relative to direct gaze, lowers mood and satisfaction of basic needs of belonging, control, self-esteem, and meaningful existence.

Thus, it is not surprising that people are highly sensitive to being attended to (i.e., looked at) by other people, especially those who have been excluded. Past research has shown that ostracized participants look more to the eyes of the interaction partner who had the power to reintegrate them, suggesting that they attempted to make eye contact to get involved into the interaction (Böckler et al., 2014). More recently, excluded participants, compared to included participants, recognized a wider range of gaze directions as being directed at them, possibly because observing direct gaze could make them feel reaffiliated (Lyyra et al., 2017).

However, as already discussed, without the perception of an opportunity for reaffiliation, ostracized individuals may begin to view other people as particularly unfriendly and start to disengage from interactions. For instance, excluded participants, compared to an inclusion group and a nonsocial control group, accepted a smaller range of gaze directions as being directed at them, probably because they do not perceive any opportunity of reconnection with others (Syrjämäki et al., 2018).

A common approach to investigate such a human predisposition to the detection of the eye gaze of others is to study attentional shifts in response to observed eye gaze direction, namely social attention. Social attention refers to the ability to orient attentional resources after observing others’ directional behavior (e.g., Friesen and Kingstone, 1998; Langton and Bruce, 1999; Nummenmaa and Calder, 2009). Research on social attention has primarily focused on the role of gaze (e.g., Frischen et al., 2007) by adopting modifications of the classical Posner’s spatial cueing paradigm (Posner et al., 1978; Posner, 1980; Posner and Cohen, 1984), namely the gaze-cueing paradigm (for a review, see Frischen et al., 2007). In a typical gaze-cueing task, an onscreen face is presented and displays averted gaze to the left or right. After a given period of time (Stimulus Onset Asynchrony, SOA), a lateral target appears at the looked at or non-looked at location. The standard approach compares detection speed for visual targets and consistently shows the gaze-cueing effect, an attentional shift toward the cued location revealed by faster responses when the target appears to the side of space that was prior cued by the gaze (Friesen and Kingstone, 1998; Driver et al., 1999; Frischen et al., 2007).

The orientation of attention in response to eye gaze provided by others appears to be rapid (e.g., Friesen and Kingstone, 1998) and reflexive (e.g., Driver et al., 1999). However, this seemingly robust and reflexive orienting response can also be sensitive to social modulators, including physical and social characteristics of the target, such as age (Ciardo et al., 2014), social status (e.g., Foulsham et al., 2010; Dalmaso et al., 2014), in-group membership and ethnicity (Pavan et al., 2011; Dalmaso et al., 2016). Moreover, individual differences of the responders can also play a role (Bayliss et al., 2005; Fox et al., 2007; Wilkowski et al., 2009). In particular, Wilkowski et al. (2009) found that individuals low in self-esteem exhibited more pronounced gaze-cueing effect than individuals high in self-esteem, and such an effect was specific to social cues. Thus, it appears that the typical tendency to orient attention in accordance with another individual’s eye gaze was enhanced under conditions of low belongingness.

## Overview of the Present Research

The present work tested whether social exclusion affects a basic cognitive phenomenon such as the gaze-cueing effect. Specifically, we investigated whether socially excluded individuals would behave differently from included ones in a gaze-cueing task.

As discussed, the social psychology literature shows multifaceted and complex responses to social exclusion. In some cases, humans show an affiliative response to exclusion (e.g., DeWall et al., 2009; Xu et al., 2015; Lyyra et al., 2017), especially when they perceive an opportunity for reaffiliation. In others, when opportunities for affiliation are not foreseen, humans may withdraw from the interactions (Romero-Canyas et al., 2010; Cuadrado et al., 2015). Thus, it is worth to address how situational factors can influence the effects of exclusion.

The gaze-cueing paradigm allows disentangling between these two competing dynamics in the context of gaze following. Thus, whether the reconnection motivation prevails, one might expect to find a larger gaze-cueing effect for socially excluded compared to socially included participants; in other words, socially excluded individuals would follow more the agent’s gaze (*Hypothesis 1a)*. On the other hand, as averted gaze may represent a further sign of social exclusion for rejected participants (Williams et al., 1998; Wirth et al., 2010) when observing faces portraying averted gaze, individuals may consider the context as lack of affiliative opportunity and wish to disengage from it. Hence, following this rationale, one might expect that excluded participants would show a reduced gaze-cuing effect than the socially included ones (*Hypothesis 1b*).

To test our hypotheses, we carried out two experimental studies in which participants were either socially excluded or included using the *Cyberball manipulation* (Williams and Blair, 2006), a paradigm in which participants engage in a ball-tossing game with virtual avatars including or excluding them from the game. In Study 1, after the Cyberball manipulation, participants performed a gaze-cueing task in which an individual’s gaze, oriented rightward or leftward, preceded a peripheral target stimulus requiring a simple categorization response. The gaze direction could be congruent or incongruent with the location of the target. In Study 2, human gazes were replaced by arrows, well-known directional nonsocial cues typically used as control for gaze in many social attention studies (e.g., Ristic et al., 2002; Friesen et al., 2004).

The procedures were approved by the Ethical Committee of the University of Milano-Bicocca, and were in accordance with the ethical standards of the 1964 Declaration of Helsinki and with the ethical standards recommended by the Italian Association of Psychology (AIP).

## Study 1

### Participants

An *a priori* power analysis was conducted for sample size estimation (using G Power 3.1; Faul et al., 2007). With an *α* = 0.05 and power = 0.95, the projected sample size needed to detect a medium effect size (*f* = 0.25) is *N* = 54 for a mixed repeated-measures ANOVA. We advertised the study, and we enrolled all the individuals who answered the call and volunteered to participate even if the final number of participants exceeded the number suggested by the G-Power analysis. Thus, 81 participants (*M_age_* = 22.90, *SD_age_ =* 1.94, range = 19–31 years, 41 females), naive to the purpose of the study, took part in the study. All participants were Italian citizens except for one Ecuadorian and one Chinese with native knowledge of the Italian language. All participants signed a form of informed consent.

### Procedure

Upon arrival at the lab, participants provided written informed consent and were asked sociodemographic information (gender, age, nationality). Next, participants were told that the study was composed of two parts, apparently unrelated to each other. In the first part, they were told that they would engage in a mental visualization task (for a similar procedure, see Williams et al., 2000). Actually, they were involved in a standard manipulation of inclusionary status. Participants played a virtual online ball-tossing game with virtual avatars, namely the Cyberball (Williams and Blair, 2006). They were told they were playing with two other players, allegedly real participants that were playing Cyberball in another lab. The three of them would take turns tossing a ball to each other. In reality, the two computer avatars were pre-programmed agents randomly assigned to either include or exclude the real participant from the ball-tossing game. In the exclusion condition, after two passes, the two computer players stopped tossing the ball to the real participant for the rest of the game. In the inclusion condition, the computer players threw the ball to the actual participant for 10 of the 30 total tosses (Williams et al., 2000).

After playing Cyberball, as a manipulation check, all participants in all conditions were asked how often (0–100% of the time) they received the ball and to report how excluded (“*I felt excluded*”) and ignored (“*I felt ignored*”) they experienced during the mental visualization task (i.e., playing Cyberball). Afterward, participants completed the Need-Threat Scale (Williams, 2009), which assessed participants’ satisfaction levels for belongingness (e.g., “*I felt rejected*”), self-esteem (e.g., “*I felt liked*”), control (e.g., “*I felt powerful*”), and meaningful existence (e.g., “*I felt invisible*”). All items were rated on 10-point scales (1 = *not at all* to 10 = *extremely*). We averaged the 20 items and created an overall index of basic needs satisfaction (*α* = 0.91). Finally, they were asked to report their current emotional state (the Rejection-related Emotions Scale, RES; Buckley et al., 2004; *α* = 0.90). The scale includes 24 items assessing 6 clusters of emotions: anger, anxiety, sadness, hurt, rejection, and happiness. Items ranged from 1 = *not at all* to 10 = *very much*. We averaged the 24 items and created an overall index of negative emotions (after reversing happiness scores; *α* = 0.90).

**Figure 1:**
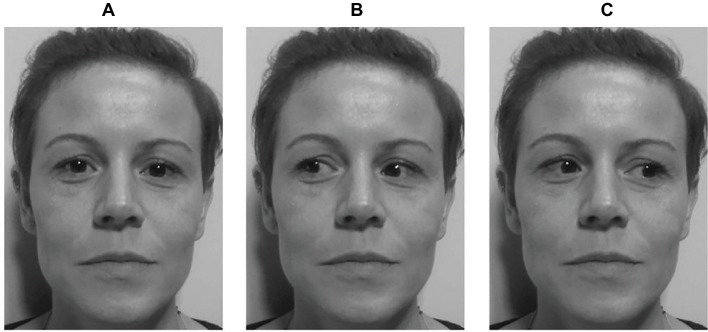
Example of a cue stimulus in Study 1. Three versions of an unfamiliar face were produced, one with gaze straight ahead **(A)**, one with the pupils averted leftward **(B)**, and another with the pupils averted rightward **(C)**. Written informed consent was obtained from the depicted individuals for the publication of their identifiable images.

The second part of the study consisted of a gaze-cueing task. The experiment was carried out in a dimly illuminated room. Participants sat approximately 76 cm away from a 22-inch LCD monitor (Asus VW226TL; resolution: 1,680 × 1,050 pixels; refresh rate: 59 Hz; horizontal screen angle: 35°31′0.82″; vertical screen angle: 20°26′0.23″) interfaced with a PC (Pentium 4). The stimuli used consisted of pictures (14.25° × 16.47°) on a gray background of unfamiliar faces gazing at different positions (about 30° left, 30° right or straight ahead—Figure 1). The distance between the two outer corners of the eyes was about 8.28°.

**Figure 2:**
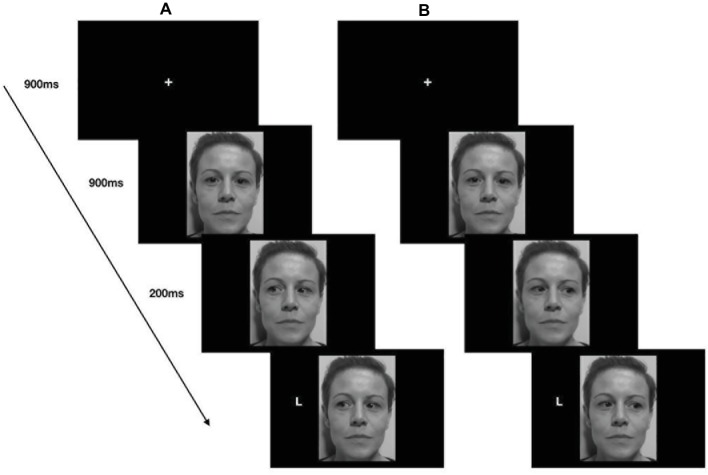
Stimuli, trial sequence, and timing of the gaze-cueing task (Study 1). Example of a congruent trial **(A)** and an incongruent trial **(B)**.

A trail started with a centrally presented fixation cross for 900 ms, which participants fixated, and then a face with direct gaze appeared in the center of the screen. After 900 ms, the eyes moved to the left or right, and 200 ms later, the target appeared either on the left or on the right of the screen, namely in a spatially congruent or spatially incongruent position with respect to the gaze direction (Figure 2).

The target remained visible until a response was made. Note that gaze direction was randomized and non-predictive of the target location. Indeed, in 50% of the trials, the target appeared in the gazed-at location, and in the other 50% of trials, the target appeared in the opposite gazed-at location. In the same vein, the target had the same probability of appearing on the right or on the left throughout each block.

Participants were asked to make speeded categorization of the target, pressing with the thumb of their (right or left) dominant hand and the forefinger (index) of the same hand the “h” key when an “L” appeared on the screen or the space-bar key when a “T” appeared. Since the “h” key is directly above the space bar, such up/down response was orthogonal to the left/right target location. Responses were allowed after the letter appearance and reaction times (RTs) were recorded.

The experimental session was composed of 8 training trials followed by 1 block of 128 experimental trials. The order of trials was randomized. At the end of the gaze-cueing task, participants were fully debriefed.

Hence, the experimental design consisted of a 2 (social exclusion manipulation: excluded vs. included) × 2 (cue-target spatial congruency: congruent vs. incongruent) factorial design, with the first factor varying between participants and the second factor varying within participants.

### Results

#### Inclusionary Status Manipulation Checks

A series of independent-samples *t*-tests revealed that excluded participants reported receiving fewer tosses (*M* = 5.05, *SD* = 3.23) than included participants (*M* = 29.92, *SD* = 9.58), *t*(79) = 16.04, *p* < 0.001, *d* = 3.57. Moreover, participants in the social exclusion condition felt to be more excluded (*M* = 7.07, *SD* = 3.10) than included participants did (*M* = 2.00, *SD* = 1.58), *t*(79) = −9.08, *p* < 0.001, *d* = −2.02, and more ignored (*M* = 7.79, *SD* = 2.62) than included participants did (*M* = 2.13, *SD* = 1.74), *t*(79) = −11.30, *p* < 0.001, *d* = −2.52.

Additionally, participants who were excluded reported higher level of rejection-related emotions (*M* = 4.12, *SD* = 1.44) than included participants (*M* = 2.90, *SD* = 0.90), *t*(79) = −4.50, *p* < 0.001, *d* = −1.00, and lower level of basic needs satisfaction (*M* = 4.08, *SD* = 1.40) than those who were included (*M* = 5.99, *SD* = 1.22), *t*(79) = 6.50, *p* < 0.001, *d* = 1.45.

#### Gaze-Cueing Task

We excluded from the analysis training trials (5.88% of trials) and errors (e.g., pressing the “h” key when a “T” appeared; 4.35% of trials). Mean and RTs are reported in millisecond (ms).

First, we conducted an error analysis. Thus, a 2 (cue-target spatial congruency: congruent vs. incongruent) × 2 (social exclusion manipulation: excluded vs. included) mixed-factors ANOVA was carried out on the percentage of the errors. Neither main effects nor interaction effects were significant, *p >* 0.29.

**Figure 3:**
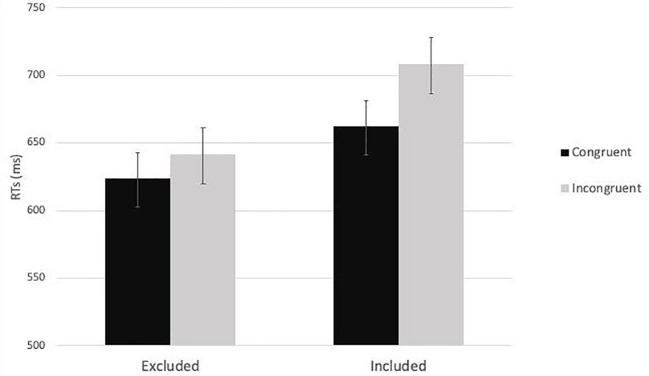
The two-way interaction effect between social exclusion manipulation and cue-target spatial congruency in Study 1.

Then, a 2 (cue-target spatial congruency: congruent vs. incongruent) x 2 (social exclusion manipulation: excluded vs. included) mixed-factors ANOVA was carried out on the average RTs. As expected, the analysis showed a significant main effect of cue-target spatial congruency, *F*(1,79) = 32.28, *p <* 0.001, *η*_p_^2^ = 0.29. RTs for congruent trials (*M* = 642.99, *SE* = 14.28) were faster than RTs for incongruent trials (*M* = 674.82, *SD* = 15.76). The main effect of the social exclusion manipulation did not reach the level of significance, *F*(1,79) = 3.16, *p =* 0.08, *η*_p_^2^ = 0.04. Importantly, the interaction effect between social exclusion manipulation and cue-target spatial congruency was significant, *F*(1,79) = 6.30, *p =* 0.014, *η*_p_^2^ = 0.07. The *post hoc* analyses revealed, in the inclusion condition, significant differences between RTs in congruent (*M* = 662.20 ms, *SE* = 20.81 ms) and incongruent trials (*M* = 708.10 ms, *SE* = 22.96 ms), *p* < 0.001. The same pattern emerged in the exclusion condition: RTs in congruent trials (*M* = 623.79 ms, *SE* = 19.56 ms) were lower than RTs in incongruent trials (*M* = 641.55 ms, *SE* = 21.58 ms), *p* = 0.023 (Figure 3).

#### Discussion

Results of Study 1 showed faster responses in congruent trails than in incongruent trials. Consistent with the literature in the gaze-cueing effect (for a review, see Frischen et al., 2007), our findings suggest that eye gaze is likely to shift an individual’s attention.

Importantly, with regard to our hypothesis, we found a significant interaction between social exclusion manipulation and the cue-target spatial congruency. Specifically, socially excluded, compared to included, participants showed a reduced attentional shift due to averted eye-gaze detection.

Study 2 aimed to explore whether such a moderating role of social exclusion on social attention could be found also with symbolic cues. To this aim, in Study 2, we adopted a procedure similar to Study 1 with one exception. Here, we replaced human gazes with arrow cues, well-known nonsocial cues typically used as a control for gaze in social attention studies (e.g., Ristic et al., 2002; Friesen et al., 2004).

## Study 2

### Participants

As for Study 1, we advertised the study, and we enrolled all the volunteers who answered the call even if the final number of participants exceeded the number suggested by the G-Power analysis. Thus, 80 participants (*M_age_* = 25.00, *SD_age_ =* 5.10, range = 19–52 years, 58 females), naive to the purpose of the study, took part in the study. All participants were Italian citizens except for one Romanian, one Ukrainian, one French, and one Italian-French with native knowledge of the Italian language. All participants provided a written informed consent.

### Procedure

**Figure 4:**
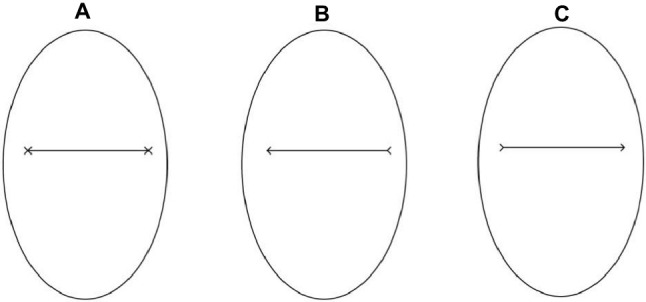
Example of a cue stimulus in Study 2: an oval framing a black segment with two Xs, at each end **(A)**, a black arrow pointing either leftward **(B)** or rightward **(C)**.

**Figure 5:**
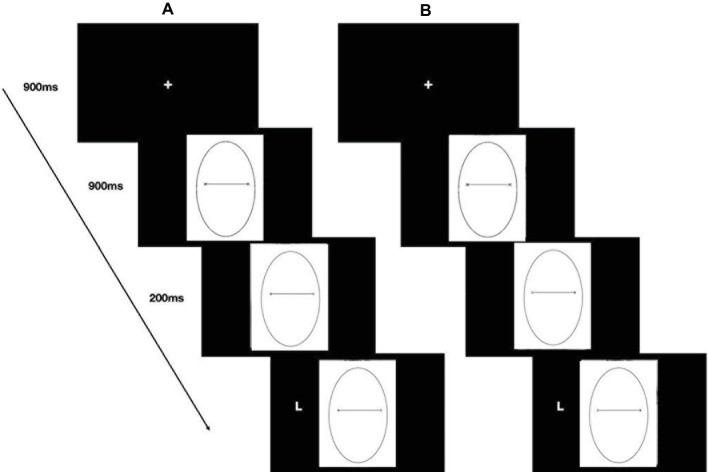
Stimuli, trial sequence, and timing of the cueing task (Study 2). Example of a congruent trial **(A)** and an incongruent trial **(B)**.

The stimuli used in Study 2 consisted of pictures (14.25° × 16.47°) of an oval of the same size as the face used in Study 1 (10.90° × 6.63°—Figure 4A) framing either a black segment with two Xs, one at each end or with a black arrow pointing either to the right or to the left. The two pointing arrows were obtained by removing half the segments of each X-endpoint (Figures 4B,C). The distance between the endpoints measured about 8.28°. The experimental procedure of Study 2 was identical to that used in the previous study (Figure 5). Hence, the experimental design consisted of a 2 (social exclusion manipulation: excluded vs. included) × 2 (cue-target spatial congruency: congruent vs. incongruent) factorial design, with the first factor varying between participants and the second factor varying within participants.

### Results

#### Inclusionary Status Manipulation Checks

A series of independent-samples *t*-tests revealed that excluded participants reported receiving fewer tosses (*M* = 8.45, *SD* = 11.18) than included participants (*M* = 32.68, *SD* = 8.48), *t*(78) = 10.92, *p <* 0.001, *d* = −2.07. Moreover, participants in the social exclusion condition felt to be more excluded (*M* = 7.83, *SD* = 2.28) than included participants did (*M* = 1.48, *SD* = 1.01), *t*(78) = −16.13, *p* < 0.001, *d* = −3.61, and more ignored (*M* = 7.90, *SD* = 1.88) than included participants did (*M* = 1.58, *SD* = 1.13), *t*(78) = −18.25, *p* < 0.001, *d* = −4.08.

Additionally, participants who were excluded reported higher level of rejection-related emotions (*M* = 4.71, *SD* = 1.30) than included participants (*M* = 2.57, *SD* = 0.68), *t*(78) = −9.26, *p* < 0.001, *d* = 2.44, and lower level of basic needs satisfaction (*M* = 3.60, *SD* = 0.91) than those who were included (*M* = 6.90, *SD* = 1.28), *t*(78) = 13.28, *p* < 0.001, *d* = 2.97.

#### Cueing Task

As in Study 1, we excluded from the analysis training trials (5.88% of trials) and errors (e.g., pressing the “h” key when a “T” appeared; 5.48% of trials). Mean and RTs are reported in millisecond (ms).

First, we conducted an error analysis. Thus, a 2 (cue-target spatial congruency: congruent vs. incongruent) × 2 (social exclusion manipulation: excluded vs. included) mixed-factors ANOVA was carried out on the percentage of the errors. Neither main effects nor interaction effects were significant, *p >* 0.43.

**Figure 6:**
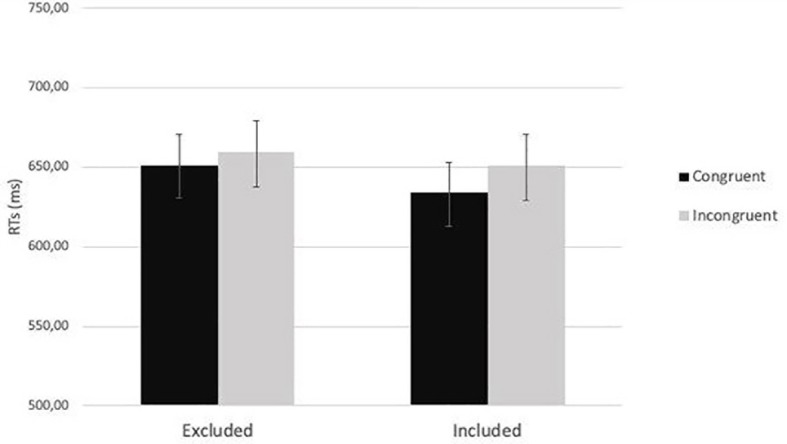
The two-way interaction between social exclusion manipulation and cue-target spatial congruency (*p* = 0.42) in Study 2.

Then, a 2 (cue-target spatial congruency: congruent vs. incongruent) × 2 (social exclusion manipulation: excluded vs. included) mixed-factors ANOVA was carried out on the average RTs. The analysis showed a significant main effect of cue-target spatial congruency, *F*(1,78) = 4.9, *p =* 0.03, *η*_p_^2^ = 0.06: RTs for congruent trials (*M* = 642.73, *SE* = 11.36) were faster than RTs for incongruent trials (*M* = 655.10, *SD* = 12.25). Neither the main effect of social exclusion manipulation, *F*(1,78) = 0.31, *p =* 0.58, *η*_p_^2^ = 0.004, nor the interaction between social exclusion manipulation and cue-target spatial congruency were significant, *F*(1,78) = 0.66, *p =* 0.42, *η*_p_^2^ = 0.008 (Figure 6).

#### Discussion

Our data revealed a cueing effect for arrow cues, namely faster RT in congruent trials than in incongruent trials. This result is in line with previous studies (e.g., Ristic et al., 2002; Tipples, 2002; Friesen et al., 2004; Quadflieg et al., 2004; Kuhn and Kingstone, 2009; Galfano et al., 2012) suggesting that, similar to gaze, arrow cues are likely to elicit strongly automatic shifts of attention.

However, results of Study 2 did not reveal any interaction effect between social exclusion manipulation and cue-target spatial congruency.

## General Discussion

The present work aimed at investigating whether social exclusion could affect the basic cognitive phenomenon of gaze-cueing effect, namely, the tendency to reorient attentional resources to the same location that other people are looking at. Specifically, we tested whether socially excluded individuals would behave differently from included individuals in a gaze-cueing task. To this end, socially excluded or included (through the *Cyberball* manipulation) participants performed a cueing task. In Study 1, they observed human faces portraying averted eye gaze; in Study 2, human gazes were replaced by arrow cues.

As previously discussed, humans respond to social exclusion in multifaceted ways. They can show affiliative response to exclusion (e.g., DeWall et al., 2009; Xu et al., 2015; Lyyra et al., 2017), especially when they perceive an opportunity for reaffiliation, or, without any opportunity for reaffiliation, they may disengage from the interactions (Romero-Canyas et al., 2010; Cuadrado et al., 2015). The paradigm adopted in the current work allowed disentangling between these two competing dynamics. Thus, if the tendency for reaffiliation prevails, one might expect to find a larger gaze-cueing effect (i.e., higher difference between RTs in congruent trials and RTs in incongruent trials) for socially excluded compared to socially included participants. That is, socially excluded individuals should follow the agent’s gaze more. On the other hand, as averted gaze may represent a further sign of social exclusion (Williams et al., 1998), individuals may consider faces portraying averted gaze as lacking affiliative opportunity. Hence, if the attentional disengagement overcomes as a reaction to exclusion, one might expect that excluded participants would show a reduced gaze-cueing effect (i.e., the lower difference between RTs in congruent trials and RTs in incongruent trials) than socially included participants.

Firstly, corroborating the idea that eye gaze (for a review, see Frischen et al., 2007) and well-known symbolic cues (e.g., Ristic et al., 2002; Tipples, 2002; Friesen et al., 2004; Quadflieg et al., 2004; Kuhn and Kingstone, 2009; Galfano et al., 2012) are capable of orienting the individual’s attention, we found faster responses in congruent trails than in incongruent trials, both when observing social (i.e., eye gaze) and symbolic cues (i.e., arrows).

Interestingly however, our data revealed a reduced attentional shift for excluded than for included participants, and such an effect was specific for social cues. Importantly, the interaction between social exclusion and cue-target spatial congruency disappeared with symbolic cues (i.e., arrows; Study 2).

Our findings are in line with the recent literature on social exclusion and eye gaze direction. For instance, Syrjämäki and Hietanen (2018) investigated whether the attentional holding effect of direct gaze would be increased for socially excluded (or included) individual. In this research, participants took part in a *Cyberball* manipulation, and, afterward, they performed an attentional disengagement task, in which they were required to identify peripheral stimuli shown to the left or right of centrally presented faces portraying direct or downward eye gaze. Results revealed that included individuals, compared to excluded individuals, disengaged slower from direct-gaze faces. Thus, social inclusion might have activated affiliation-related cognitive processes causing delayed disengagement of attention from faces cueing affiliation. In the same vein, our findings suggest that an averted gaze may represent a further sign of social exclusion, and, as a consequence, excluded participants showed a reduced cueing effect toward faces communicating a lack of affiliative opportunity.

In Study 2, participants observed symbolic cues. As in the previous study, we found the typical cueing effect, but, in this case, no interaction effect has emerged. Taken together, it seems that the moderating role of social exclusion on social attention is specific for social cues, as shown in Study 1 with eye gaze, and not generalizable to symbolic cues, as in Study 2 with arrows. Thus, given that most of the cognitive psychology literature has established the primacy of socially relevant stimuli, such as eye gaze (Langton et al., 2000), and that socially excluded individuals are particularly sensitive to social cues (e.g., Pickett et al., 2004), it appears that social exclusion may moderate social attention only when social cues are available.

Our findings are theoretically relevant for several reasons. As reported above, past research showed increased sensitivity to other people’s gaze after social exclusion. Direct gaze, for instance, indicates that another’s attention is directed to the self (Conty et al., 2016), so it may be especially significant for individuals who have been excluded. Indeed, previous studies have shown that ostracized participants, when trying to reconnect with others, seek for inclusive cues, and direct gaze represents an important social cue to this purpose.

Furthermore, because negative attention is preferred to being ignored (e.g., O’Reilly et al., 2014), even when direct gaze could be perceived as a sign of threat (e.g., when it is accompanied by an angry facial expression; Adams and Kleck, 2005), it might reduce the unpleasant effects of exclusion (Rudert et al., 2017; but see also Syrjämäki et al., 2017). Averted gaze represents a primary cue for communicating ostracism and no opportunity for reconnection with others (Williams et al., 1998) although it can signal a danger approaching or the location of an interesting object. Both laboratory and field studies (in social psychology) demonstrate that averted eye gaze from live or virtual confederates can induce feelings of ostracism and basic need threat similar to traditional social exclusion manipulations (e.g., Wirth et al., 2010; Wesselmann et al., 2012; Böckler et al., 2014). In our gaze-cueing paradigm, the observed agents, after a brief initial period of direct eye gaze, consistently portrayed averted gazes, both in congruent trails than in incongruent trials. Hence, our excluded participants can have perceived the averted gaze as a further sign of social exclusion. As noted, socially excluded people desire to make new social connections, but they also wish to make sure that they will not suffer rejection again (Park and Baumeister, 2015), so that when there is no opportunity for reaffiliation, they may withdraw from the interaction (Romero-Canyas et al., 2010; Cuadrado et al., 2015). Thus, it is plausible that, to cope with exclusion, our participants showed reduced cueing effect toward such faces signaling a lack of affiliative opportunity.

Moreover, our findings allow us to disentangle between different alternative explanations. As mentioned, prior research showed that social exclusion deploys cognitive resources (Chester and DeWall, 2014; Lurquin et al., 2014; Riva et al., 2014a). Hence, whether the moderating role of social exclusion on social attention was due to the limited cognitive resources, we would have found similar results either when observing social cues (Study 1) and nonsocial cues (Study 2). Interestingly however, we did not find any interaction between congruency and social exclusion with symbolic cues (Study 2). Thus, we could interpret our findings from a motivational perspective; for socially excluded participants, symbolic cues could have served as informative cues, but not useful for the benefit of the interaction. However, since we did not manipulate the type of cue (social vs. symbolic) in the same experiment, the speculation on these different patterns requires caution and further empirical investigation.

In addition, the present work also extends the existing literature from a methodological point of view. Our results demonstrate that the gaze-cueing paradigm is an appropriate task that helps to shed light on two competing dynamics occurring during social exclusion, namely the reaffiliation and the disengagement strategies.

There are some limitations to the present research to be considered that can be addressed in future research. It is worth noting that the stimuli adopted here were much simpler than situations we face in everyday life. Indeed, the stimuli used in the current experiments were static photographs of an individual appearing on a computer screen. We speculate that in more ecological contexts (e.g., observations of people in real social interactions), social exclusion could even enhance the effect on social attention, probably because real averted gaze may represent stronger signs of rejection. Hence, further research should explore the relationship between social attention and social exclusion using more ecological paradigms, for instance showing real interactions or dynamic displays of eye gaze.

From a methodological perspective, here we conducted two different studies, the first focused on the role of eye gaze, and the second focused on the role of arrow. Direct comparisons between social and nonsocial cues may enrich our knowledge on whether such a moderating role of social exclusion on social attention is specific for social cues or generalizable to symbolic cues. For instance, further research should compare in the same experiment both the role of eye gaze and arrow (i.e., either with a within-participants design whereby both arrow and eye gaze are presented in the same experiment or with a between-participants design), in order to replicate and extend these results.

Furthermore, the current study did not involve a control group that was neither included nor excluded. Thus, we are not fully able to disentangle whether the observed differences between excluded and included groups are due to exclusion, inclusion, or both. However, previous studies found that being included in Cyberball has similar psychological consequences to watching a mountain sketch on the screen (Riva et al., 2014b). Nevertheless, future studies should consider avoiding this problem, for instance, by employing a nonsocial control group (see for instance Syrjämäki et al., 2018).

To sum up, the current work illustrated that social exclusion modulates the basic cognitive process of social attention; following exclusion, individuals showed reduced gaze-cueing effect toward faces portraying averted gaze, which represents a further sign of rejection.

## Ethics Statement

The procedures were approved by the Ethical Committee of the University of Milano-Bicocca, and were in accordance with the ethical standards of the 1964 Declaration of Helsinki and with the ethical standards recommended by the Italian Association of Psychology (AIP). All subjects gave written informed consent.

## Author Contributions

RC drafted the first version of the manuscript and ran the studies. RC and PRiv conducted data analyses. PRiv, PRic, and SS conceived the study idea and the experimental paradigm, and supervised and coordinated the research. PRiv, PRic, and SS reviewed the final version of the manuscript.

### Conflict of Interest Statement

The authors declare that the research was conducted in the absence of any commercial or financial relationships that could be construed as a potential conflict of interest.
